# Effect of Qiling Jiaogulan Powder on Pulmonary Fibrosis and Pulmonary Arteriole Remodeling in Low-Temperature-Exposed Broilers

**DOI:** 10.3390/ani13010005

**Published:** 2022-12-20

**Authors:** Juan Yu, Peng Li, Zhibian Duan, Xingyou Liu

**Affiliations:** 1 School of Life Sciences and Basic Medicine, Xinxiang University, Xinxiang 453003, China; 2 College of Veterinary Medicine, Shanxi Agricultural University, Taigu 030800, China

**Keywords:** Qiling Jiaogulan Powder, broiler, pulmonary fibrosis, pulmonary arteriole remodeling, TGF-β1/Smad2 signaling pathway

## Abstract

**Simple Summary:**

Chinese herbal medicine contains a variety of active ingredients, and it is a multifunctional feed additive with nutritional and pharmacological properties. It is a potential alternative antibiotic product in the commercial production of poultry. Our study demonstrated that Qiling Jiaogulan Powder improved pulmonary fibrosis and pulmonary arteriole remodeling, reduced the occurrence and development of pulmonary arterial hypertension induced by low-temperature exposure in broilers, and played a positive role in reducing the mortality and culling rate of broilers.

**Abstract:**

Chinese herbal medicine plays an important role in regulating the nutritional metabolism of poultry and maintaining or improving normal physiological functions and animal health. The present study investigated the effects of dietary supplementation with Qiling Jiaogulan Powder (QLJP) on pulmonary fibrosis and pulmonary arteriole remodeling in low temperature-exposed broilers. Seven-day-old Ross 308 broilers (*n* = 240) were reared adaptively to 14 days of age. The broilers were randomly divided into six groups: A control group (basal diet and normal feeding temperature); model group (basal diet); low-, medium- and high-dose QLJP groups (basal diet supplemented with 1 g/kg, 2 g/kg, 4 g/kg QLJP); and L-Arg group (basal diet supplemented with 10 g/kg L-arginine). Additionally, all the broilers, except the broilers in the control group, from the age of 14 days old, had a house temperature continuously lowered by 2 °C each day until it reached 12 °C at 21 days of age, and the low temperature was maintained until the end of the experiment. There were four replicates per group and 10 birds per replicate. The results showed that the structure of the lung tissue was clearer and basically intact in the broilers in the QLJP groups, with a small number of collagen fibers formed, and the content of hydroxyproline (HYP) was significantly reduced. QLJP improved pulmonary arteriole lesions, such as tunica media thickening, intimal hyperplasia, arterial wall hypertrophy, and lumen narrowing. QLJP reduced the relative media thickness (%) and relative medial area (%) of the pulmonary arteriole, and significantly decreased the expression level of the alpha-smooth muscle actin (α-SMA) protein in pulmonary arteriole, which alleviated pulmonary arteriole remodeling. The quantitative real-time PCR (qPCR) and enzyme-linked immunosorbent assay (ELISA) results showed that QLJP treatment significantly reduced the gene and protein levels of transforming growth factor-beta l (TGF-β1) and Smad2 in the lung and downregulated the gene and protein levels of collagen type I alpha 1 (COL1A1) and matrix metalloproteinase 2 (MMP2). In conclusion, the results of our study suggested that dietary supplementation with QLJP improved pulmonary fibrosis and pulmonary arteriole remodeling by inhibiting the expression of genes related to the TGF-β1/Smad2 signaling pathway and inhibited the occurrence and development of pulmonary arterial hypertension in low-temperature-exposed broilers.

## 1. Introduction

Low ambient temperature is one of the most common environmental stressors during broiler farming. Low-temperature exposure causes an increased metabolic rate and oxygen demand in broilers, and the lung and cardiovascular system are highly stressed, which increases oxidative stress and tissue damage in broilers and leads to hypoxia. The hypoxic response causes lung endothelial injury or dysfunction, which increases the cell proliferation and extracellular matrix synthesis that contribute to pulmonary fibrosis and pulmonary vascular remodeling and leads to pulmonary arterial hypertension (PAH) [1,2,3]. PAH in broilers is a hypoxia problem caused by stress factors, such as low ambient temperature. PAH leads to substandard slaughter weights and a large increase in mortality and culling rates. It causes massive economic losses in the chicken industry [4,5]. Pulmonary vascular remodeling is an important pathological feature of PAH and may progressively increase pulmonary artery pressure and pulmonary vascular resistance and lead to culling and death [6,7]. Screening for drugs to improve pulmonary lesions and pulmonary vascular remodeling in broilers remains an important measure for the treatment of PAH in broilers.

Traditional Chinese medicine may be used as a feed additive because of its wide sources, variety and natural ingredients that improve the resistance of poultry to stress and diseases. These treatments show great developmental potential in preventing and reducing diseases [8,9].

Qiling Jiaogulan Powder (QLJP) is a classic traditional Chinese medicine formula recorded in the Veterinary Pharmacopoeia of the People’s Republic of China for the treatment of nutritional and metabolic diseases in broilers [10]. QLJP consists of five herbs: Radix Astragalus (*Astragalus membranaceus* (Fisch.) Bunge), Poria (*Poria cocos* (Schw.) Wolf), Radix Arnebiae (*Arnebia euchroma* (Royle) Johnst), Herba Gynostemmae (*Gynostemma pentaphyllum* (Thunb.) Makino), and Rhizoma Alismatis (*Alisma orientale* (Sam.) Juzep). Modern pharmacological studies showed that *Astragalus membranaceus* had antioxidant activity and significantly improved lung function and pulmonary vascular remodeling [11,12,13,14]. *Poria cocos* has anti-inflammatory and antioxidant functions, and it improved fibrotic damage induced by the TGF-β/Smad signaling pathway [15,16]. *Arnebia euchroma* has anti-inflammatory and antioxidant activity, and it regulates cardiovascular activity, inhibits the TGF-β/Smad signaling pathway and alleviates fibrotic damage [17,18,19]. *Gynostemma pentaphyllum* has anti-inflammatory and antioxidant effects, and it reduces PAH [20,21,22]. *Alisma orientale* improves lung inflammation and injury and vascular function, exhibits antioxidant activity, and lowers blood pressure [23,24,25].

Activation of the TGF-β1 signaling pathway predicts the occurrence of organ fibrosis in many diseases [26]. Activation of the TGF-β1/Smad2 signaling pathway resulted in a large deposition of the extracellular matrix components, such as collagen fibers, which induced pulmonary fibrosis [27]. The TGF-β1/Smad2/3 signaling pathway increased the proliferation of pulmonary smooth muscle cells and decreased apoptosis, which resulted in pulmonary arterial remodeling [28,29]. Collagen, such as COL1A1, one of the major structural components in the extracellular matrix of the lung parenchyma, vessels play an important role in hypoxia-induced pulmonary vascular remodeling [30]. A-SMA is involved in endothelial mesenchymal transformation and proliferation of vascular adventitia fibroblasts and plays an important role in vascular remodeling [31,32]. Chen et al. showed that activation of the TGF-β1/Smad2 signaling pathway promoted fibroblast transformation to a proliferative phenotype and adventitial fibrosis by upregulating **α**-SMA and COL1A1, which led to pulmonary arterial remodeling and PAH [33]. Matrix metalloproteinases (MMPs) are involved in extracellular matrix metabolism and are closely related to pulmonary arterial remodeling. An increase in MMP activity and abnormal metabolism of the extracellular matrix promoted the occurrence of PAH in an experimental model [34,35]. Activation of MMP-2/9 in the lung participated in the deposition of the extracellular matrix induced by the TGF-β1/Smad2 pathway and promoted pulmonary arterial remodeling [36]. The TGF-β1/Smad2 signaling pathway is an important regulatory pathway for pulmonary fibrosis and PAH, and it is involved in many cardiovascular diseases [37,38]. However, the role of the TGF-β1/Smad2 signaling pathway in the development of broiler PAH is not clear and needs further investigation.

Based on the rich chemical composition of QLJP and its multidirectional pharmacological activities, the present study investigated the effect of QLJP on pulmonary fibrosis and pulmonary arteriole remodeling in low-temperature-exposed broilers and elucidated the related mechanism.

## 2. Materials and Methods

### 2.1. Preparation of QLJP

Radix Astragalus (Huangqi, product lot number: 21081202), Poria (Fuling, product lot number: 201101), Radix Arnebiae (Zicao, product lot number: 200201), Herba Gynostemmae (Jiaogulan, product lot number: 200501), and Rhizoma Alismatis (Zexie, product lot number: 210901) were obtained from Bozhou Zhang Zhongjing Chinese Herbal Pieces Co., Ltd. (Anhui, China). According to the method in the Veterinary Pharmacopoeia of the People’s Republic of China [10], the five herbs and proportions in QLJP were prepared (Table 1). The five herbs were weighed, ground into a fine powder and mixed to obtain QLJP.

### 2.2. Analysis of QLJP Using Ultrahigh Phase Liquid Chromatography–Mass Spectrometry (UHPLC–MS)

Four grams of QLJP was weighed, extracted with 80% methanol, and filtered through a 0.22 μm microporous membrane to obtain the sample to be tested. Ultra-high-pressure liquid chromatography was performed using a Thermo Scientific Vanquish UHPLC system. The following chromatography conditions were as used: Thermo ScientificTM Hypersil Gold Vanquish (2.1 × 100 mm, 1.9 µm); column temperature, 30 °C; injection volume, 5 µL; and flow rate, 0.3 mL/min. The following gradient elution procedure with mobile phase A (H_2_O with 0.1% formic acid) and mobile phase B (acetonitrile) was used: 0~2 min, 10%~10% B; 2~10 min, 10%~50% B; 10~13 min, 50%~80% B; 13~14 min, 80%~95% B; 14~14.1 min, 95%~10% B; and 14.1~18 min, 10%~10% B.

Thermo ScientificTM Q ExactiveTM Plus was used for mass spectrometry analysis. The following ion source properties were used: Positive and negative ion detection patterns; spray voltage, 3.5 kV (+), 3.2 kV (−); sheath gas flow rate, 35 arbitrary units; Aux gas flow rate, 15 arbitrary units; capillary temperature, 320 °C; and Aux gas heater temp, 350 °C. The following scan settings were used for full MS: resolution, 70,000; AGC target, 1 × 10^6^; and maximum IT, 100 ms. The scan settings for dd-MS2 were as follows: Resolution, 17,500; AGC target, 2 × 10^5^; maximum IT, 50 ms; and isolation window, 1.6 m/z.

### 2.3. Animal Experimental Design

All experiments were approved and performed in accordance with the guidelines of the Animal Ethics Committee of the Henan Institute of Science and Technology (LLSC 2022024/2022-3). Seven-day-old Ross 308 broilers (*n* = 240) were adaptively reared until 14 days of age. The broilers were randomly divided into six groups: A control group (Control); model group (Model); low-, medium- and high-dose QLJP groups (Low, Medium, and High); and positive control group (L-arginine, L-Arg). There were four replicates per group and 10 birds per replicate. All broilers were allowed to drink and eat freely. The broilers in the control group were fed a basal diet and tap water, and the house temperature was gradually lowered to approximately 25 °C from 14 days of age until the end of the experiment. All broilers, except the broilers in control group, from the age of 14 days old, the house temperature was continuously lowered by 2 °C each day until it reached 12 °C at 21 days of age, and the low temperature was maintained until the end of the experiment. From 14 to 42 days of age, the QLJP groups were fed a basal diet mixed with QLJP (high, medium, and low: 4 g/kg, 2 g/kg, 1 g/kg) [10]. The positive control group was supplemented with 10 g/kg L-arginine in the basal diet (Tianjin Jinyao Amino Acid Co., Ltd., Tianjin, China) [39].

### 2.4. Sample Collection

Five broilers were randomly selected from each group at 21, 28, 35, and 42 days of age, respectively. The broilers of all experimental groups were sacrificed via cervical dislocation. The lung were rapidly separated and washed with precooled normal saline. Part of the lung tissue was transected along the left lung hilum and fixed in a 4% paraformaldehyde solution for histopathological analysis with Masson’s trichrome staining and Weigert-Van Gieson staining. Another part of the lung tissue was frozen in liquid nitrogen and stored at −80 °C for later use.

### 2.5. Masson’s Trichrome Staining of the Lung

Lung tissues were processed into routine paraffin sections with a thickness of 5 μm, followed by Masson’s trichromatic staining (Masson’s Trichrome Stain Kit, Solarbio Science and Technology Co., Ltd., Beijing, China). Stained sections were quickly dehydrated with 95% ethanol for 3 s and dehydrated three times with absolute ethanol for 10 s each. The sections were cleared twice with xylene for 1 min each time, and the slides were sealed with neutral gum. Morphological changes in the lung were observed under an optical microscope (Nikon Eclipse ci, imaging system: Nikon DS-FI2).

### 2.6. Determination of the Hydroxyproline Content in the Lung

According to the instructions of the hydroxyproline (HYP) content detection kit (Solarbio Science and Technology Co., Ltd., Beijing, China), the absorbance value of lung tissue hydrolysate was measured at 560 nm, and the HYP content in the lung was calculated.

### 2.7. Determination of Pulmonary Arteriole Remodeling

Routine 5 μm paraffin sections of the lung were prepared and stained according to the instructions of the Weigert-Van Gieson staining solution (Solarbio Science and Technology Co., Ltd., Beijing, China). Under an optical microscope, elastic fibers were blue–black, collagen fibers were red, and other components in the background were yellow. Pulmonary arterioles with diameters of 50 to 100 μm and 100 to 200 μm were analyzed using Image-Por Plus 6.0 image analysis software. The external and inner diameter of pulmonary arterioles, vessel wall area and vascular total area were measured, and the relative media area (%) and relative media thickness (%) of the pulmonary arterioles were calculated according to the method of Barth [40] and used to evaluate pulmonary arteriole remodeling [41].

### 2.8. Immunohistochemical Analysis

The protein expression of α-SMA in the pulmonary arterioles was detected using immunohistochemistry (IHC). The primary antibody was an α-SMA mouse monoclonal antibody (Santa Cruz, CA, USA), and the secondary antibody was a horseradish peroxidase (HRP)-conjugated goat anti-mouse IgG (Absin Biotechnology Co., Ltd., Shanghai, China). Routine IHC steps were performed, and sites positive for the expression of α-SMA were brown–yellow under a microscope. Pulmonary arterioles were used for the evaluation of α-SMA protein expression in the pulmonary arterioles. Image-Pro Plus 6.0 software was used to determine the average optical density (AOD) of α-SMA positivity. Seven pulmonary arterioles with similar diameters were randomly selected from each slice for measurement, and the average value was taken as the representative value of the slice.

### 2.9. Quantitative Real-Time PCR Detection

Lung tissue was removed from the −80 °C refrigerator and thoroughly triturated in liquid nitrogen. Total RNA was extracted from the lung tissue using TRIzol (Invitrogen, CA). Reverse transcription was performed according to the instruction manual of the PrimeScript RT Reagent Kit with the gDNA Eraser Reverse Transcription Kit, and the cDNA was stored at −20 °C for future use. Quantitative real-time PCR (qPCR) was performed using TB Green Premix Ex Taq II (Takara Biomedical Technology Co., Ltd., Dalian, China), and the expression levels of *TGF-β1*, *Smad2*, *COL1A1* and *MMP2* mRNA were detected, *β-actin* was used as an internal reference gene. Each sample was repeated at least three times. The 2^−ΔΔCt^ method was used to calculate the relative quantitative expression of the target gene in each sample. The primer sequences are shown in Table 2.

### 2.10. ELISA Detection

One hundred milligrams of lung tissue was added to 100 µL of precooled PBS (pH 7.4) to prepare a tissue homogenate using a low-temperature homogenizer. The homogenate was centrifuged at 3000 rpm for 20 min, and the supernatant was collected for detection. There were five replicates per group, and each sample had three repetitions. ELISAs were performed according to the instructions of the Chicken transforming growth factor β1 (TGF-β1) ELISA Kit, Chicken Smad2 (Smad2) ELISA Kit, Chicken collagen alpha-1(I) chain (COL1A1) ELISA Kit, and Chicken matrix metalloproteinase 2 (MMP2) ELISA Kit (Enzyme-linked (Mlbio) Biotechnology Co., Ltd., Shanghai, China). The double antibody sandwich ELISA method was used to detect the protein levels of TGF-β1, Smad2, COL1A1 and MMP2.

### 2.11. Statistical Analysis

Data are expressed as the mean ± SEM. SPSS 23.0 statistical software was used for one-way ANOVA followed by the LSD post hoc test. Differences were considered statistically significant at the level of *p* < 0.05.

## 3. Results

### 3.1. Identification of the Components in QLJP using UHPLC-MS

**Figure 1:**
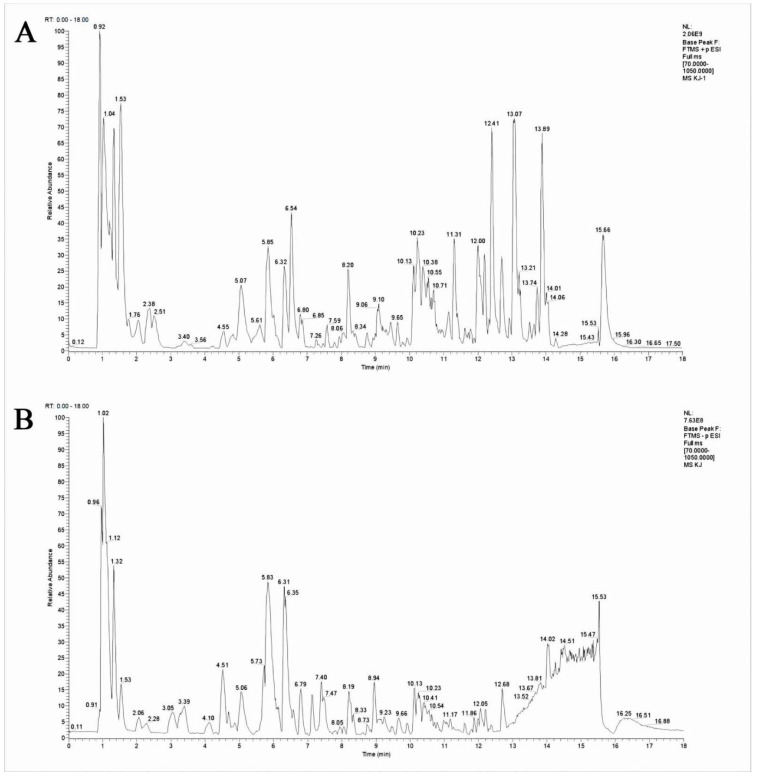
The total ion chromatogram of QLJP. (**A**) positive ion mode and (**B**) negative ion mode.

The components of QLJP were analyzed using UHPLC–MS. The total ion chromatogram of QLJP was obtained in negative and positive ion modes (Figure 1). Comparison with the reference compounds in the database revealed 27 classes of constituent compounds of QLJP, including carboxylic acids and derivatives, organooxygen compounds, flavonoids, steroids and steroid derivatives, macrolides and analogs, peptidomimetics, phenols, linear 1,3-diarylpropanoids, benzopyrans, indoles and derivatives, organonitrogen compounds, pyrans, oxepanes, coumarins and derivatives, isoflavonoids, pyridines and derivatives, cinnamic acids and derivatives, diazanaphthalenes, lactones, macrolactams, aurone flavonoids, benzofurans, naphthalenes and others. Flavonoid compounds were identified as the main medicinal components of QLJP and included rutin, kaempferol-3-O-rutinoside, formononetin, isoquercitrin, quercetin, kaempferol, and kaempferol-3-O-glucoside (Figure 1, Supplementary Table S1).

### 3.2. The Effect of QLJP on Broiler Pulmonary Fibrosis

**Figure 2:**
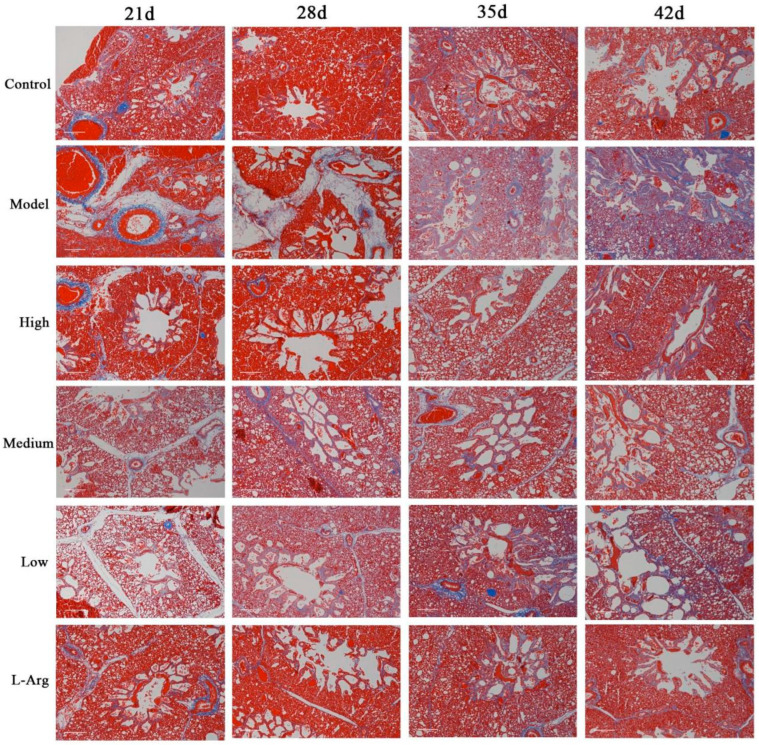
The effect of QLJP on broiler pulmonary fibrosis by Masson’s trichrome staining (100×, scale bar = 100 µm). Control is the control group, Model is the model group, High is the high dose group of QLJP, Medium is the medium dose group of QLJP, Low is the low dose group of QLJP, and L-Arg is the L-Arginine group.

Masson’s trichrome staining in the lung were observed under a microscope. Collagen fibers were blue, muscle fibers and erythrocytes were red, and cell nuclei were dark brown. The lung tissue structure in the control group was basically normal, with a small amount of blue collagen fibers. Compared to the control group, the pulmonary interstitium was significantly widened in broilers in the model group, with inflammatory cell infiltration, and the tertiary bronchi and alveolar ducts were severely dilated. At 35 d and 42 d, the alveolar wall was thickened, the lung tissue structure was disordered, some alveolar structures were destroyed, and a large number of blue collagen fibers were formed. Compared to the model group, supplementation of the basal diet with QLJP improved the lung tissue structure and reduced the degree of fibrosis. The lung structure was basically normal in the high- and medium-dose groups, with less collagen fiber formation. Additionally, the tertiary bronchi were slightly dilated and some collagen fibers had formed at 35 d and 42 d in the low-dose group. These results showed that QLJP improved the lung tissue structure and alleviated pulmonary fibrosis (Figure 2).

### 3.3. The Effect of QLJP on the HYP Content in Lung

**Figure 3:**
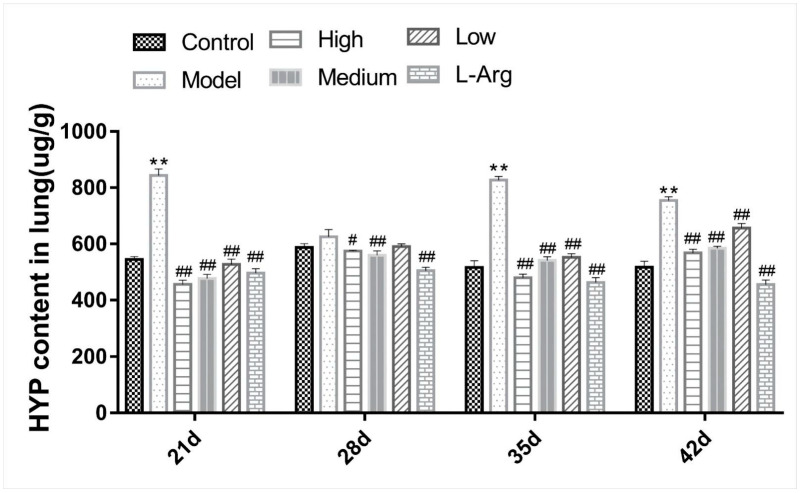
The effect of QLJP on the HYP content in lung of broilers. Data are expressed as the mean ± SEM, *n* = 5. ** *p* < 0.01, compared to the control group; ^#^ *p* < 0.05, ^##^ *p* < 0.01, compared to the model group.

The content of HYP reflects the degree of pulmonary fibrosis. The HYP content in the model group was markedly higher than the control group at 21 d, 35 d and 42 d (*p* < 0.01). Compared to the model group, the content of HYP in the medium- and high-dose QLJP groups was significantly decreased at each time point (*p* < 0.05 or *p* < 0.01)*,* and the content of HYP in the low-dose group was obviously decreased at 21 d, 35 d and 42 d (*p* < 0.01). These results further showed that supplementation of the basal diet with QLJP alleviated pulmonary fibrosis (Figure 3).

### 3.4. The Effect of QLJP on the Pathological Changes of Pulmonary Arterioles

**Figure 4:**
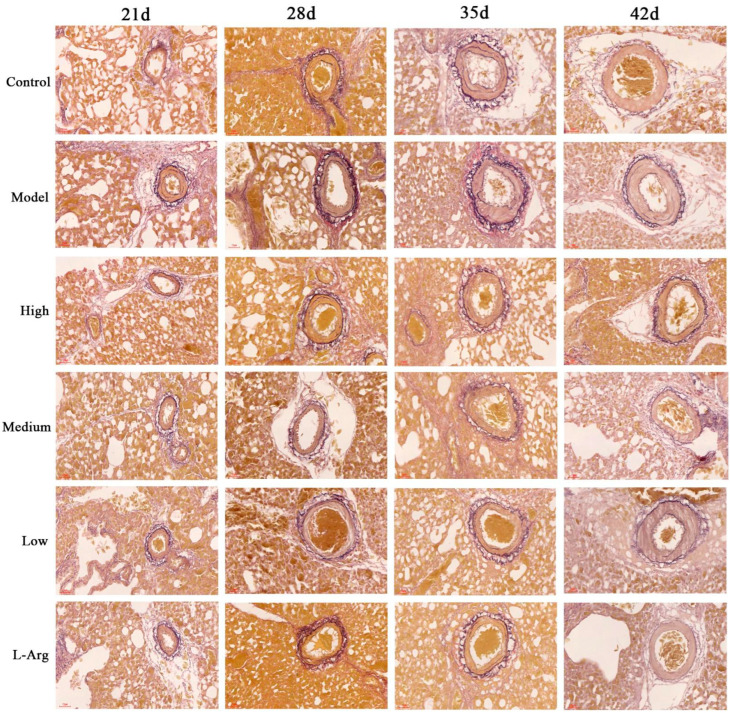
The effect of QLJP on the pathological changes of pulmonary arterioles by Weigert-Van Gieson staining (400×, scale bar = 20 µm). Control is the control group, Model is the model group, High is the high dose group of QLJP, Medium is the medium dose group of QLJP, Low is the low dose groupof QLJP, and L-Arg is the L-Arginine group.

Weigert-Van Gieson staining revealed the morphological changes of pulmonary arterioles. Elastic fibers were blue and black, collagen fibers were red, and the other components in the background were yellow. Compared to the control group at the same day of age, more collagen fibers were formed in the pulmonary arterioles in the model group, the tunica media thickened, the intima proliferated, and the lumen narrowing, which were significant at 21 d, 35 d and 42 d, more elastic fibers and collagen fibers were formed in the model group at 28 d. Compared to the model group at the same day of age, these lesions were significantly alleviated in the high-dose and medium-dose groups, and the tunica media of pulmonary arterioles was thickened at the 42 d low-dose group. These results showed that QLJP alleviated the pathological changes of pulmonary arterioles, such as tunica media thickening and lumen narrowing (Figure 4).

### 3.5. The Effect of QLJP on Pulmonary Arteriole Remodeling

**Figure 5:**
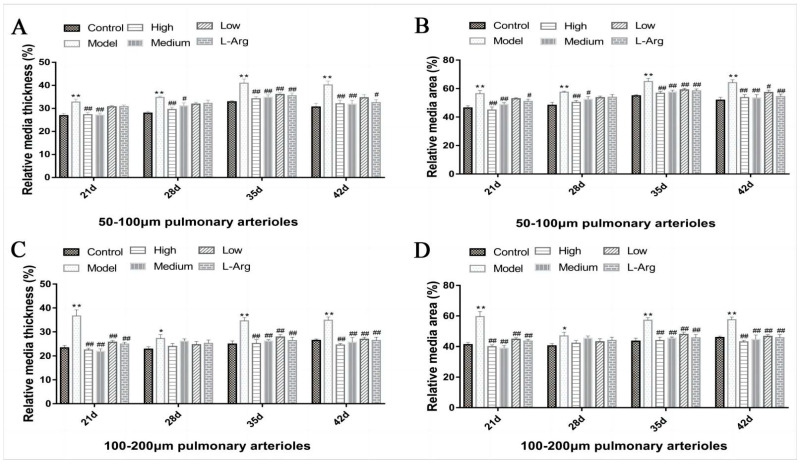
The effect of QLJP on the pulmonary arteriole remodeling of broilers. (**A**) Relative media thickness (%) of pulmonary arterioles with diameters of 50–100 µm (*n* = 6), (**B**) relative medial area (%) of pulmonary arterioles with diameters of 50–100 µm (*n* = 6), (**C**) relative media thickness (%) of pulmonary arterioles with diameters of 100–200 µm (*n* = 5), (**D**) relative medial area (%) of pulmonary arterioles with diameters of 100–200 µm (*n* = 5). Data are expressed as the mean ± SEM, * *p* < 0.05, ** *p* < 0.01, compared to the control group; ^#^ *p* < 0.05, ^##^ *p* < 0.01, compared to the model group.

The results of the analyses of the relative media thickness (%) and relative medial area (%) of pulmonary arterioles with diameters of 50–100 µm are shown in Figure 5A,B. At each time point, the relative media thickness (%) and relative medial area (%) in the model group were obviously higher than the control group (*p* < 0.01). Compared to the model group, the relative media thickness (%) and relative medial area (%) were reduced in the medium- and high-dose QLJP groups at each time point (*p* < 0.05 or *p* < 0.01). The relative media thickness (%) at 35 d and the relative medial area (%) at 35 d and 42 d in the low-dose QLJP group were significantly decreased (*p* < 0.05 or *p* < 0.01).

Figure 5C,D show the relative media thickness (%) and relative medial area (%) of pulmonary arterioles with diameters of 100–200 µm. The relative media thickness (%) and relative medial area (%) in the model group were much higher than the control group at each time point (*p* < 0.05 or *p* < 0.01). After QLJP treatment, the relative media thickness (%) and relative medial area (%) were significantly decreased at 21 d, 35 d and 42 d in the low-, medium-, and high-dose QLJP groups (*p* < 0.01).

The relative media thickness (%) and relative media area (%) of pulmonary arterioles were used to evaluate vascular remodeling. The results showed that pulmonary arteriole remodeling occurred in the model group, and QLJP inhibited pulmonary arteriole remodeling induced by low-temperature exposure in broilers.

### 3.6. The Effect of QLJP on the Level of a-SMA Protein in Pulmonary Arterioles by IHC

**Figure 6:**
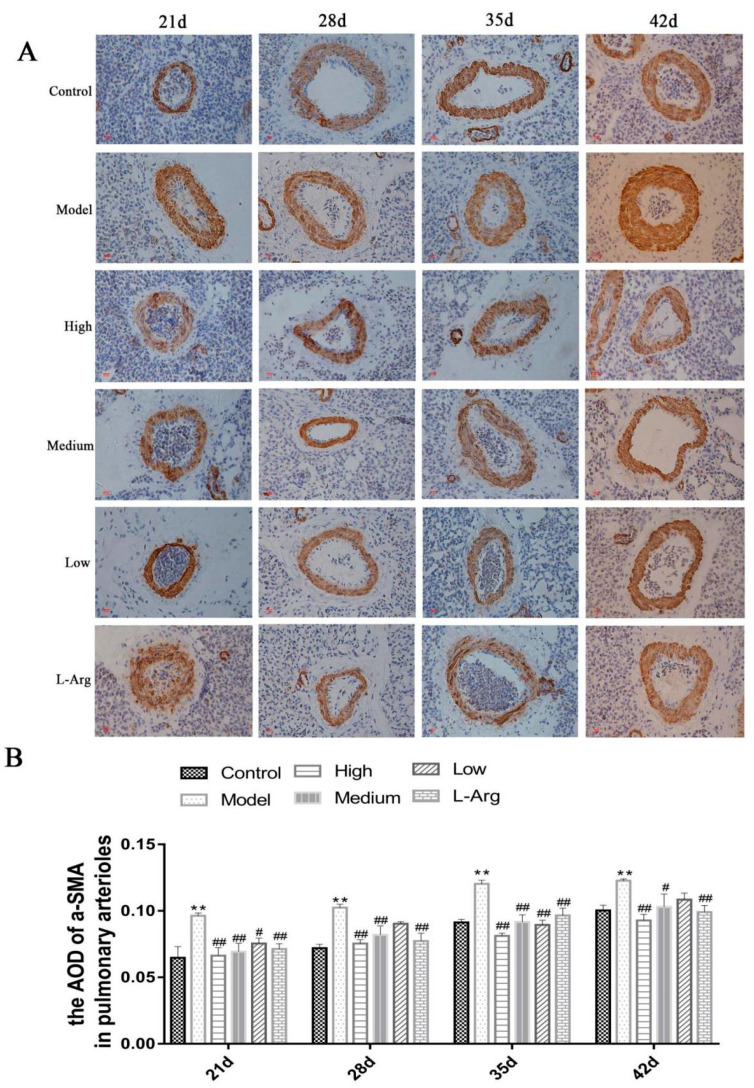
The effect of QLJP on the level of α-SMA protein in pulmonary arterioles by IHC. (**A**) Representative images of α-SMA in pulmonary arterioles by IHC (400×, scale bar = 20 µm), (**B**) the average optical density of α-SMA in pulmonary arterioles. Data are expressed as the mean ± SEM, *n* = 7. ** *p* < 0.01, compared to the control group; ^#^ *p* < 0.05, ^##^ *p* < 0.01, compared to the model group.

The expression level of α-SMA protein in pulmonary arterioles was calculated as described in the experimental methods. At each time point, the protein expression levels of α-SMA in the model group were significantly increased compared to the control group (*p* < 0.01). Compared to the model group, the protein expression levels of α-SMA were obviously decreased in the medium- and high-dose QLJP groups at each time point and at 21 d and 35 d in the low-dose QLJP group (*p* < 0.05 or *p* < 0.01). The IHC results showed that supplementation of basal diet with QLJP decreased the protein expression levels of α-SMA in pulmonary arterioles, which was beneficial for inhibiting pulmonary arteriole remodeling (Figure 6).

### 3.7. QLJP Reduced the Expression Levels of TGF-β1 and Smad2 in the Lung

**Figure 7:**
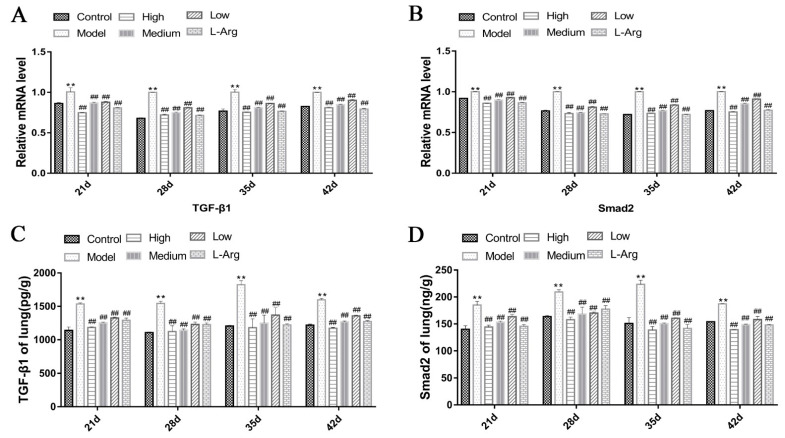
QLJP reduced the expression levels of TGF-β1 and Smad2 in the lung. (**A**) Relative mRNA level of *TGF-β1*, (**B**) relative mRNA level of *Smad2*, (**C**) the protein level of TGF-β1, and (**D**) the protein level of Smad2. Data are expressed as the mean ± SEM, *n* = 5. ** *p* < 0.01, compared to the control group; ^##^ *p* < 0.01, compared to the model group.

As shown in Figure 7A,B, the expression levels of *TGF-β1* and *Smad2* mRNA in the model group were much higher than the control group at each time point (*p* < 0.01). Compared to the model group, the expression levels of *TGF-β1* and *Smad2* mRNA were significantly decreased in the low-, medium- and high-dose QLJP groups at each time point (*p* < 0.01). The ELISA results presented in Figure 7C,D show that the protein levels of TGF-β1 and Smad2 in the model group were obviously higher than those in the control group at each time point (*p* < 0.01). However, the protein levels of TGF-β1 and Smad2 were significantly reduced in the QLJP treatment groups at each time point compared to the model group (*p* < 0.01). These results showed that QLJP inhibited the activity of the TGF-β1/Smad2 signaling pathway.

### 3.8. QLJP Downregulated the Expression Levels of COL1A1 and MMP2 in the Lung

**Figure 8:**
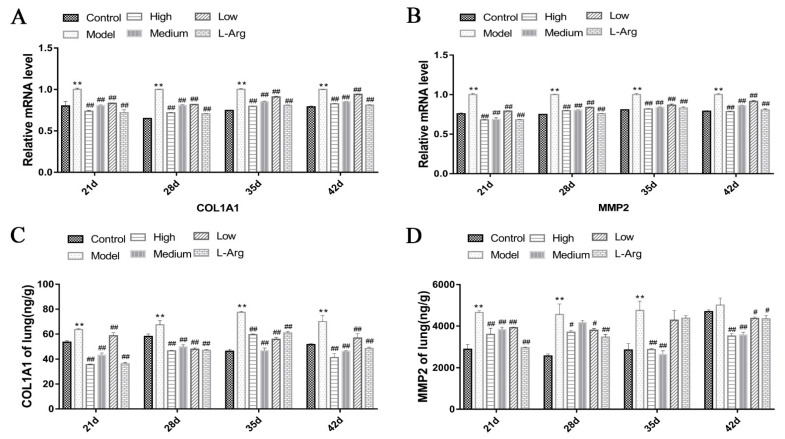
QLJP downregulated the expression levels of COL1A1 and MMP2 in the lung. (**A**) Relative mRNA level of *COL1A1*, (**B**) relative mRNA level of *MMP2,* (**C**) the protein level of COL1A1, (**D**) the protein level of MMP2. Data are expressed as the mean ± SEM, *n* = 5. ** *p* < 0.01, compared to the control group; ^#^ *p* < 0.05, ^##^ *p* < 0.01, compared to the model group.

The qPCR results are shown in Figure 8A,B. The expression levels of *COL1A1* and *MMP2* mRNA were significantly higher in the model group than the control group at each time point (*p* < 0.01). Compared to the model group, the expression levels of *COL1A1* and *MMP2* mRNA were obviously lower in the QLJP-treated groups at each time point (*p* < 0.01). Figure 8C,D show COL1A1 and MMP2 protein levels in the lung. Compared to the control group, the protein level of COL1A1 at each time point and the protein level of MMP2 at 21 d, 28 d and 35 d were significantly higher in the model group (*p* < 0.01). However, the protein levels of COL1A1 in the low-, medium- and high-dose QLJP groups were much lower than the model group at each time point (*p* < 0.01). QLJP reduced the protein level of MMP2 at each time point in the high-dose group (*p* < 0.05 or *p* < 0.01), at 21 d, 35 d and 42 d in the medium-dose group (*p* < 0.01), and at 21 d, 28 d and 42 d in the low-dose group (*p* < 0.05 or *p* < 0.01). These results showed that QLJP reduced the expression levels of COL1A1 and MMP2.

## 4. Discussion

The disharmony between the rapid growth and development of various organs in broilers leads to an increased susceptibility to PAH [42]. Low-temperature exposure increases the metabolic rate and oxygen requirement of broilers, which exacerbates hypoxia [43]. Hypoxia causes a series of pathological symptoms, such as destruction of the lung parenchyma structure, pulmonary vascular remodeling and right heart hypertrophy, which lead to PAH. PAH adversely affects survival and contributes to impaired functional capacity [44,45]. Pulmonary vascular remodeling in PAH is closely related to pulmonary fibrosis, and PAH aggravates pulmonary fibrosis and pulmonary vascular remodeling [46]. Traditional Chinese medicine is used to treat pulmonary diseases, and it protects pulmonary vascular endothelial cells, inhibits vascular inflammation, inhibits the proliferation and migration of pulmonary vascular cells, and inhibits pulmonary vascular remodeling via multi-target and multi-pathway effects to play an important role in the prevention and treatment of PAH [9,47,48].

PAH aggravates the load of the right heart, reduces heart function, obstructs blood circulation throughout the body, causes congestion and edema in lung and other tissues to further aggravate tissue hypoxia stress and leads to extensive pathological damage to organs and tissues [49,50]. In our study, clinical and autopsy observations and related index tests found that broilers had the symptoms or lesions such as pulmonary edema, heart failure and ascites (These data have been applied in future publication). Masson’s trichrome staining of the lung in broilers showed that the pulmonary interstitium was significantly widened in the model group, with a large number of infiltrated inflammatory cells. The tertiary bronchi and alveolar ducts were severely dilated, the structure of the lung was disordered, and a large number of collagen fibers were deposited. The HYP content in the lung of the model group was higher than the control group, which also indicated that more collagen fibers formed in the lungs of the model group. The pulmonary fibrosis injury of broilers in the model group in our study was consistent with the finding that activation of the TGF-β1/Smad2-dependent pathway causes pulmonary fibrosis, as reported by Maxwell [51].

Pulmonary vascular remodeling is an important pathological feature of PAH. Thickening of the pulmonary media, hyperplasia of the intima and lumen stenosis are the main characteristics of pulmonary vascular remodeling [52]. Our Weigert-Van Gieson staining and imaging analysis of pulmonary arterioles were consistent with Yang et al. [41], which indicated that pulmonary vascular remodeling occurred in the pulmonary arterioles in the model group. A-SMA is a molecular marker of pulmonary arterial myofibroblast activation. The expression of α-SMA in pulmonary artery is increased in animal models of PAH, and it promotes the proliferation of pulmonary artery smooth muscle cells and causes thickening of the pulmonary artery media, which lead to pulmonary artery remodeling [53,54]. The IHC results for pulmonary arterioles α-SMA in the model group were consistent with these findings. In summary, our experiment results indicated that the low temperature-induced PAH model in broilers was successful.

QLJP is a classic traditional Chinese medicine formula composed of *Astragalus membranaceus*, *Poria cocos*, *Arnebia euchroma*, *Gynostemma pentaphyllum*, and *Alisma orientale*. *Astragalus membranaceus* extract inhibits the process of pulmonary fibrosis induced by the TGF-β1/Smad signaling pathway and has good efficacy against pulmonary fibrosis [55]. Quercetin is an important flavonoid component of *Astragalus membranaceus* and *Gynostemma pentaphyllum*, and it reduces collagen secretion and inhibits α-SMA expression via the TGF-β1/Smad2/3 signaling pathway to play an antifibrotic role [56]. Wang et al. showed that triterpenoids from *Poria cocos* inhibited the TGF-β1/SMAD2 signaling pathway and played an antifibrotic role [57]. Compared to the model group, the structure of the lung tissue was basically clear after QLJP treatment, the deposition of collagen fibers was reduced, and the HYP content in the lung was obviously decreased, which also indicated that QLJP had anti-pulmonary fibrosis properties.

Astragalus polysaccharides have a protective effect on hypoxia-induced PAH in mice by reducing the protein levels of TGF-β1, α-SMA, VEGF and HIF-1α, reducing the collagen fiber deposition area of pulmonary artery and inhibiting pulmonary artery remodeling [58]. Quercetin improved hypertension-induced vascular remodeling in hypertensive rats by reducing aortic oxidative stress and MMP2 activity [59]. Compound Poria Linglicorice decoction improved pulmonary artery remodeling by inhibiting the expression of TGF-β1 in the pulmonary artery of rats and effectively reduced PAH [60]. Gypenosides regulate the expression of TGF-β1 and its related receptor pathways SMADs, α-SMA, COL1A1, TGF-βR1, TGF-βR2 and other targets to inhibit activation of the TGF-β/Smad2/3 signaling pathway [61]. The present study found that QLJP reduced the formation of pulmonary arteriole collagen fibers, improved wall hypertrophy, lumen narrowing and other lesions, decreased the relative media thickness (%) and relative medial area (%) of pulmonary arterioles, and downregulated the protein expression level of α-SMA in pulmonary arterioles. These results indicated that QLJP inhibited pulmonary arteriole remodeling in broilers. The gene and protein levels of TGF-β1 and Smad2 in the lung were significantly decreased, and the gene and protein levels of COL1A1 and MMP2 were significantly downregulated in the low-, medium- and high-dose QLJP groups. These qPCR and ELISA results were consistent with the results of Wu et al. [62], which further demonstrated that QLJP played a pharmacological role in the prevention and treatment of pulmonary fibrosis and pulmonary arteriole remodeling induced by low-temperature exposure in broilers. In addition, our results demonstrated that low-temperature exposure induced PAH in the model group to cause weight loss of broilers in the model group, with a significantly higher feed-to-weight ratio than the other experimental groups. QLJP treatment could inhibit the occurrence of PAH, improve the body weight of broilers and reduce the feed-to-weight ratio than the model group. These data have been applied in future publication.

## 5. Conclusions

Our results showed that the TGF-β1/Smad2 signaling pathway was involved in broiler PAH by regulating pulmonary fibrosis and pulmonary arteriole remodeling. However, QLJP inhibited the TGF-β1/Smad2 signaling pathway, down-regulated the expression levels of α-SMA, COL1A1, MMP2, and improved pulmonary fibrosis and pulmonary arteriole remodeling induced by low-temperature exposure to play a role in the prevention and treatment of PAH. For the first time, this study investigated the pharmacological mechanism of QLJP in preventing and treating broiler PAH by regulating the TGF-β1/Smad2 signaling pathway, which provides a scientific basis for QLJP as a potential feed additive to reduce the mortality and culling rate of broilers.

## Figures and Tables

**Table 1:** The compositions of QLJP.

English Name	Latin Scientific Name	Parts Used	Origin	Amount (g)
Radix Astragalus	*Astragalus membranaceus* (Fisch.) Bunge	Radix	Inner Mongolia	100
Poria	*Poria cocos* (Schw.) Wolf	Peeled sclerotia	Yun Nan	75
Radix Arnebiae	*Arnebia euchroma* (Royle) Johnst	Radix	Yili, Xinjiang	75
Herba Gynostemmae	*Gynostemma pentaphyllum* (Thunb.) Makino	Herba	Shiyan, Hubei	175
Rhizoma Alismatis	*Alisma orientale* (Sam.) Juzep	Rhizoma	Si Chuan	75

**Table 2:** Primers for qPCR.

Gene Name	Primer Sequences
*TGF-β1*	F 5′-CCGATGAGTATTGGGCCAAAGAGC-3′ R 5′-GACACGTTGAACACGAAGAAGATGC-3′
*Smad2*	F 5′-TGTCATCCATTCTGCCATTCACTCC-3′ R 5′-CACCACTTCTCCTCTTGCCCATTC-3′
*COL1A1*	F 5′-TGGATTCTCGGTTACTGCTGTTGATAG-3′ R 5′-TTCGGGTTTCCACACATCCTTATCG-3′
*MMP2*	F 5′-CAACAGAAGGCAGGACAGATGGATAC-3′ R 5′-GGAAGATGAAGGGGAATACACAAGGAG-3′
*β-actin*	F 5′-CATCTATGAAGGCTACGC-3′ R 5′-GGCTGTGGTGGTGAAG-3′

## Data Availability

The data sets during and analyzed during the current study are available from the corresponding author on reasonable request.

## Supplementary Materials

The following supporting information can be downloaded at: https://www.mdpi.com/article/10.3390/ani13010005/s1, Table S1: Flavonoids compounds of QLJP.
