# Supplementary material for: Effect of Qiling Jiaogulan Powder on Pulmonary Fibrosis and Pulmonary Arteriole Remodeling in Low-Temperature-Exposed Broilers
Source: Animals (Basel). 2022 Dec 20;13(1):5. doi: 10.3390/ani13010005 (PMC9817788; doi:10.3390/ani13010005)
Supplement: Supplementary file 1 [file animals-13-00005-s001.zip › animals-1982153-supplementary.pdf]

Supplementary Table S1

Flavonoids compounds of QLJP.

Compound ID	Class	Subclass	Name	Formula	Annot Delta Mass ppm	Calc MW	Area	RT/min
m0653	Flavonoids	Flavonoid glycosides	Rutin	$C_{27}H_{30}O_{16}$	-1.53	610.15245	9962104197	5.845
m0767	Flavonoids	Flavonoid glycosides	Kaempferol-3-O-rutinoside	$C_{27}H_{30}O_{15}$	-0.99	594.15788	6427538706	6.336
m1680	Isoflavonoids	O-methylated isoflavonoids	Formononetin	$C_{16}H_{12}O_{4}$	1.6	268.07399	729816079.4	10.121
m0702	Flavonoids	Flavonoid glycosides	Isoquercitrin	$C_{21}H_{20}O_{12}$	0.91	464.0959	660441915.8	6.089
m1277	Flavonoids	Flavones	Quercetin	$C_{15}H_{10}O_{7}$	-0.73	302.04243	656770372.6	8.239
m1508	Flavonoids	Flavones	kaempferol	$C_{15}H_{10}O_{6}$	-1.46	286.04732	581520787.9	9.274
m0826	Flavonoids	Flavonoid glycosides	kaempferol-3-O-glucoside	$C_{21}H_{20}O_{11}$	0.16	448.10063	483046354.5	6.595
m0822	Flavonoids	Flavonoid glycosides	Astragalin	$C_{21}H_{20}O_{11}$	0.15	448.10063	468506099.4	6.587
m0652	Flavonoids	Flavonoid glycosides	quercetin-3-O-beta-glucopyranoside	$C_{21}H_{20}O_{12}$	0.2	464.09557	405584404.4	5.844
m1119	Isoflavonoids	Isoflavonoid O-glycosides	Ononin	$C_{22}H_{22}O_{9}$	0.23	430.12648	362383547.5	7.625
m1200	Flavonoids	Flavonoid glycosides	Demethoxycentaureidin 7-O-rutinoside	$C_{29}H_{34}O_{16}$	-0.47	638.18438	262762296.1	7.936
m0274	Flavonoids	Flavans	Taxifolin	$C_{15}H_{12}O_{7}$	1.04	304.05862	162068438	2.996
m1380	Flavonoids	Flavonoid glycosides	Pectolinarin	$C_{29}H_{34}O_{15}$	-0.33	622.18956	145743092.7	8.647
m1862	Flavonoids	Flavones	chrysin	$C_{15}H_{10}O_{4}$	0.95	254.05815	143302274.5	11.203
m1521	Flavonoids	Flavones	isorhamnetin	$C_{16}H_{12}O_{7}$	1.36	316.05873	136149840.4	9.38
m0851	Flavonoids	Flavonoid glycosides	Isorhamnetin 3-galactoside	$C_{22}H_{22}O_{12}$	0.01	478.11113	108919705	6.676
m0849	Flavonoids	Flavonoid glycosides	isorhamnetin-3-O-glucoside	$C_{22}H_{22}O_{12}$	0.13	478.11119	106382118.5	6.673
m1408	Flavonoids	Flavonoid glycosides	Licuroside	$C_{26}H_{30}O_{13}$	-0.52	550.16835	95774990.07	8.761
m1303	Isoflavonoids	O-methylated isoflavonoids	Formononetine	$C_{16}H_{12}O_{4}$	-3.85	268.07253	40553636.64	8.333
m0651	Flavonoids	Flavonoid glycosides	Maritimein	$C_{21}H_{20}O_{11}$	0.16	448.10063	40145691.17	5.842
m0999	Flavonoids	Flavonoid glycosides	Diosmetin-7-O-rutinoside	$C_{28}H_{32}O_{15}$	-0.1	608.17406	35559503.82	7.231
m2001	Flavonoids	Flavans	Pilosanol B	$C_{28}H_{30}O_{10}$	0.15	526.18397	34146800.34	11.694

Supplementary Table S1 Continue

Compound ID	Class	Subclass	Name	Formula	Annot Delta Mass ppm	Calc MW	Area	RT/min
m1304	Isoflavonoids	O-methylated isoflavonoids	7-hydroxy-3-(3-hydroxy-4-methoxyphenyl)-6-methoxychromen-4-one	$C_{17}H_{14}O_6$	1.33	314.07945	32430953.08	8.336
m1011	Flavonoids	Flavonoid glycosides	Phloridzin	$C_{21}H_{24}O_{10}$	-0.44	436.13676	30318646.23	7.276
m0765	Flavonoids	Flavonoid glycosides	Kaempferol-3-O-alpha-L-rhamnoside	$C_{21}H_{20}O_{10}$	-0.16	432.10558	25773983.61	6.332
m0998	Flavonoids	Flavonoid glycosides	Diosmetin-7-O-neohesperidoside	$C_{28}H_{32}O_{15}$	-0.56	608.17378	23057569.6	7.223
m1602	Isoflavonoids	O-methylated isoflavonoids	Retusin 7-methyl ether	$C_{17}H_{14}O_5$	1.33	298.08452	22145089.92	9.779
m0910	Flavonoids	Flavonoid glycosides	Homobutein 4-glucoside	$C_{22}H_{24}O_{10}$	-0.66	448.13665	21258629.57	6.904
m0892	Flavonoids	Flavonoid glycosides	Peonidin-3-O-beta-galactoside	$C_{22}H_{22}O_{11}$	0.23	462.11632	20233558.1	6.856
m1641	Flavonoids	Flavonoid glycosides	Ikarisoside E	$C_{26}H_{26}O_{10}$	-0.72	498.15224	20037723.77	9.948
m0873	Flavonoids	Flavonoid glycosides	Zosterin	$C_{25}H_{28}O_{12}$	-0.9	520.15761	16342141.84	6.786
m0708	Flavonoids	Flavonoid glycosides	Liquiritin	$C_{21}H_{22}O_9$	0.52	418.1266	15459547.88	6.105
m1764	Flavonoids	Flavans	Amoricin	$C_{31}H_{36}O_6$	-3.84	504.24925	14683216.05	10.648
m0820	Isoflavonoids	O-methylated isoflavonoids	biochanin A	$C_{16}H_{12}O_5$	-3.89	284.06737	12480583.6	6.583
m1091	Flavonoids	Flavonoid glycosides	Isorhamnetin-3-O-rutinoside	$C_{28}H_{32}O_{16}$	-0.61	624.16865	11803663.46	7.51
m0513	Flavonoids	Flavonoid glycosides	Marginatoside	$C_{33}H_{40}O_{20}$	-0.49	756.21092	11787042.47	5.277
m0781	Flavonoids	Flavonoid glycosides	6-Methoxyluteolin 7-glucoside	$C_{22}H_{22}O_{12}$	0.13	478.11119	11543115.37	6.431
m0958	Flavonoids	Flavonoid glycosides	Lantanoside	$C_{25}H_{26}O_{12}$	-0.74	518.14204	11029574.68	7.098
m0913	Flavonoids	Flavonoid glycosides	Apigenin-7-O-neohesperidoside	$C_{27}H_{30}O_{14}$	-0.47	578.16328	10168473.93	6.912
m0909	Flavonoids	Flavonoid glycosides	naringenin-7-O-glucoside	$C_{21}H_{22}O_{10}$	-0.58	434.12105	10036787.32	6.897
m0891	Flavonoids	Flavonoid glycosides	Kaempferol-3-O-alpha-L-arabinoside	$C_{20}H_{18}O_{10}$	0.47	418.09019	9868612.53	6.855
m1090	Flavonoids	Flavonoid glycosides	Subulin	$C_{28}H_{32}O_{16}$	-0.16	624.16893	9812039.01	7.51

Supplementary Table S1 Continue

Compound ID	Class	Subclass	Name	Formula	Annot Delta Mass ppm	Calc MW	Area	RT/min
m0872	Flavonoids	Flavonoid glycosides	6-Methoxyluteolin 7-glucuronide methyl ester	$C_{23}H_{22}O_{13}$	0.47	506.10628	9454043.109	6.785
m0505	Flavonoids	Flavonoid glycosides	Flavaprin	$C_{26}H_{30}O_{10}$	-0.63	502.18358	9419889.735	5.24
m1850	Flavonoids	Flavonoid glycosides	Diffutin	$C_{23}H_{28}O_{10}$	0.22	464.16835	9254711.872	11.14
m0905	Flavonoids	Flavonoid glycosides	Monospermoside	$C_{21}H_{22}O_{10}$	0.06	434.12132	9251515.462	6.89
m1239	Flavonoids	Flavans	Liquiritigenin	$C_{15}H_{12}O_4$	1.02	256.07382	8683202.74	8.098
m1040	Flavonoids	Flavonoid glycosides	Luteolin 3'-methyl ether 7-malonylglucoside	$C_{25}H_{24}O_{14}$	-0.74	548.1162	8614045.055	7.368
m0532	Flavonoids	Flavonoid glycosides	8-Hydroxyluteolin 8-glucoside-3'-rutinoside	$C_{33}H_{40}O_{21}$	1.16	772.20711	7946115.197	5.332
m0509	Flavonoids	Flavonoid glycosides	6-Hydroxyluteolin 3'-methyl ether 7-sophoroside	$C_{28}H_{32}O_{17}$	0.2	640.16408	7313854.398	5.256
m1555	Flavonoids	Hydroxyflavonoids	7-Hydroxyflavan	$C_{15}H_{14}O_2$	1.23	226.09966	7017916.717	9.589
m1363	Flavonoids	Flavonoid glycosides	Chrysin 6-C-glucoside-8-C-alpha-L -arabinopyranoside	$C_{26}H_{28}O_{13}$	-0.04	548.15297	6766257.587	8.579
m1197	Flavonoids	Flavonoid glycosides	Matteucinol 7-O-glucoside	$C_{24}H_{28}O_{10}$	-0.3	476.1681	6669362.79	7.923
m1392	Flavonoids	Flavonoid glycosides	4'-Hydroxychalcone 4'-glucoside	$C_{21}H_{22}O_7$	-0.63	386.13631	6628012.064	8.702
m0920	Flavonoids	Flavonoid glycosides	Davidioside	$C_{21}H_{24}O_9$	0.82	420.14238	6240268.582	6.927
m1065	Flavonoids	Flavonoid glycosides	Sulfurein	$C_{21}H_{20}O_{10}$	-0.87	432.10527	5273557.405	7.454
m0780	Flavonoids	Flavonoid glycosides	Leptosin	$C_{22}H_{22}O_{11}$	-0.1	462.11617	5080242.43	6.427
m0786	Flavonoids	Flavonoid glycosides	6-Hydroxyluteolin 6-xyloside	$C_{20}H_{18}O_{11}$	0.72	434.08522	4301260.226	6.458
m0624	Flavonoids	Flavonoid glycosides	6-Hydroxyluteolin 7-sambubioside	$C_{26}H_{28}O_{16}$	-0.29	596.13756	4122757.28	5.697
m0747	Flavonoids	O-methylated flavonoids	Hesperetin	$C_{16}H_{14}O_6$	1.18	302.07939	3804687.603	6.256
m1419	Flavonoids	Flavonoid glycosides	Bidenoside B	$C_{24}H_{30}O_{10}$	-0.86	478.18349	3140929.249	8.844
m1206	Flavonoids	Flavonoid glycosides	Plumerubroside	$C_{24}H_{30}O_{12}$	1.3	510.17439	2377753.244	7.942
